# The Microbiome in Pancreatic Cancer-Implications for Diagnosis and Precision Bacteriophage Therapy for This Low Survival Disease

**DOI:** 10.3389/fcimb.2022.871293

**Published:** 2022-05-19

**Authors:** Mwila Kabwe, Stuart Dashper, Joseph Tucci

**Affiliations:** ^1^Department of Rural Clinical Sciences, La Trobe Rural Health School, La Trobe University, Bendigo, VIC, Australia; ^2^La Trobe Institute for Molecular Science, La Trobe University, Bendigo, VIC, Australia; ^3^Melbourne Dental School, University of Melbourne, Melbourne, VIC, Australia

**Keywords:** bacteriophage, pancreatic cancer, microbiome, *Porphyromonas gingivalis*, oncobacterium

## Abstract

While the mortality rates for many cancers have decreased due to improved detection and treatments, that of pancreatic cancer remains stubbornly high. The microbiome is an important factor in the progression of many cancers. Greater understanding of the microbiome in pancreatic cancer patients, as well as its manipulation, may assist in diagnosis and treatment of this disease. In this report we reviewed studies that compared microbiome changes in pancreatic cancer patients and non-cancer patients. We then identified which bacterial genera were most increased in relative abundance across the oral, pancreatic, duodenal, and faecal tissue microbiomes. In light of these findings, we discuss the potential for utilising these bacteria as diagnostic biomarkers, as well as their potential control using precision targeting with bacteriophages, in instances where a causal oncogenic link is made.

## Introduction

**Figure 1:**
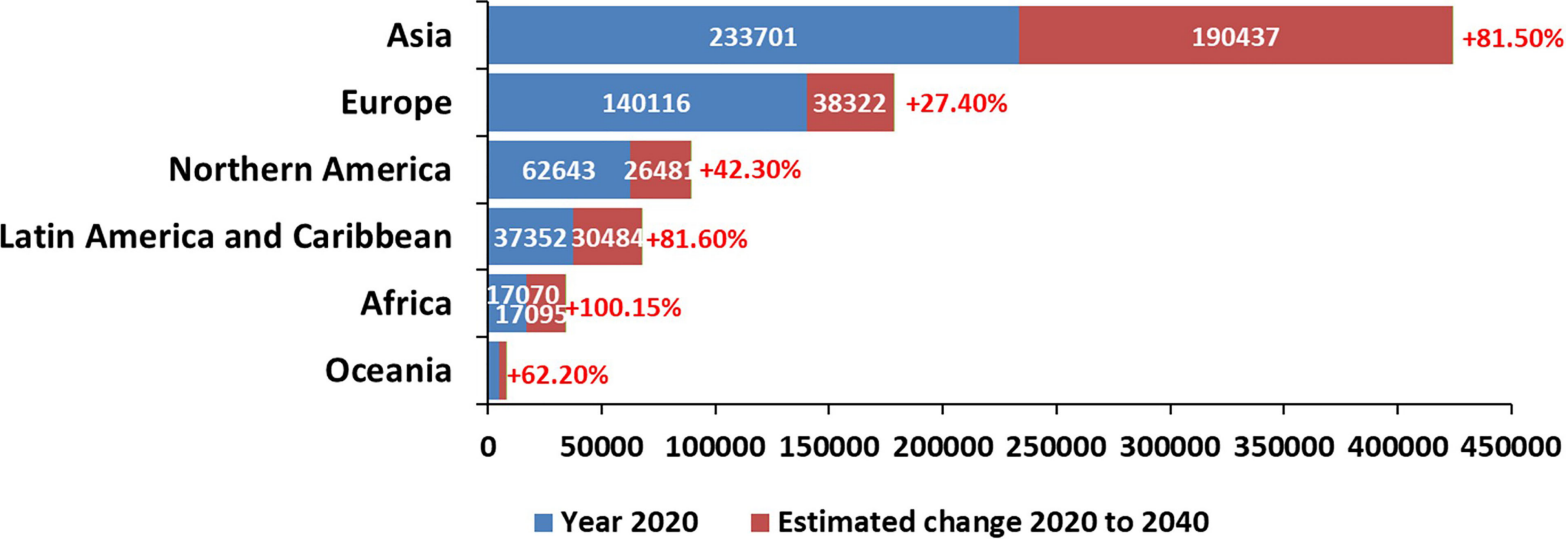
Current and projected worldwide estimations of pancreatic cancer cases (Reproduced from https://gco.iarc.fr/).

Cancer is the leading cause of death world-wide and an ever-increasing barrier to improving life expectancy (Bray et al., 2021). Due to low-survival and poor outcomes in the past decade, pancreatic cancer has been identified as the emerging cancer of most concern as it remains the only major cancer type in the European Union and the UK showing no signs of an overall fall in mortality trends (Carioli et al., 2021). While it has a relatively low incidence rate globally, pancreatic cancer is the seventh leading cause of cancer-related death and remains a lethal disease. Despite the advances in anti-cancer therapies, patients with pancreatic cancer have poor survival prospects with a five-year survival rate of just 10% (Dahan et al., 2021). Worldwide, there were 495,773 new diagnoses of pancreatic cancer in 2020 and 466,003 deaths (Sung et al., 2021). The highest incidence of pancreatic cancer is found across Europe, with new diagnoses ranging from 7-9 cases per 100 000 population. The lowest numbers are seen in parts of Africa and South-Central Asia, where incidence is recorded to be between 1-4 cases per 100, 000 population (Rawla et al., 2019; Sung et al., 2021). **Figure 1** depicts estimated numbers of cases in the world in 2020, with predictions to 2040, for pancreatic cancer.

In pancreatic cancer, surgery remains the curative treatment. However, 90% of the patients with stage I and stage II disease relapse and do not survive (Hartwig et al., 2013). While adjuvant chemotherapy (gemcitabine with erlotinib or 5 – fluorouracil and cisplatin) and radiotherapy have been evaluated in treatment of pancreatic cancer, these regimes remain controversial, and without additional survival benefits, compared to surgery (Moore, 2005; Dahan et al., 2010; Hammel et al., 2016). Current research is underway into minimally invasive endoscopic deliveries such as ^125^I seeds (Jin et al., 2008), celiac neurolysis (Wyse et al., 2011), and fiducial placement for stereotactic body radiation therapy (Sanders et al., 2010). Immunotherapy phase I/II clinical trials have failed to show any positive effects in pancreatic cancer (Kaur et al., 2021). Therefore, pancreatic cancer remains difficult to treat. As such, more extensive research into biomarkers, new precision medicine, and multidisciplinary approaches, is required to optimise therapy for individual patients (Zhu et al., 2018).

Since Warren and Marshall’s pioneering work linking *Helicobacter pylori* (initially described as a spiral or curved bacilli related to *Campylobacter* spp.) with gastric cancer (Marshall and Warren, 1984), there has been an expansion of research investigating the roles of bacteria in oncogenesis and promotion of carcinoma growth. To date bacterial species have been implicated in the initiation or progression of a wide range of different types of cancer. Recently oral bacteria including *Fusobacterium nucleatum* have been linked to cancers distant from the oral cavity (Abed et al., 2020; Parhi et al., 2020). *F. nucleatum* and other oral bacterial species such as *Prevotella heparinolytica* and *Peptostreptococcus anaerobius* are now widely accepted as oncobacteria (Brennan and Garrett, 2019, Kabwe et al., 2021a, Rai et al., 2021).

Like many cancers, early diagnosis for pancreatic cancer is important for better treatment efficacy and prognosis (Yamada et al., 2021). However, only 8% of pancreatic cancer patients are initially diagnosed with localised resectable disease (De La Cruz et al., 2014). Screening and surveillance is recommended using endoscopic ultrasound (EUS) and MRI/magnetic retrograde cholangiopancreatography (Goggins et al., 2020). EUS coupled with fine needle aspiration (EUS-FNA) provides a more definitive diagnosis with the benefits of a biopsy (Cherian et al., 2010). However, for many asymptomatic patients with localised disease cytology examinations are usually negative (Masuda et al., 2022). Despite this, the cytology samples may provide an unexplored opportunity for microbiome sequencing and use of bacterial species as biomarkers for diagnosis.

Since minimally invasive EUS-FNA are incorporated into routine diagnostics, it is possible that applying genomic screening of the tumour microbiome may offer higher resolution than cytology as well as opportunities for treatment by manipulating the microbiome using specific antimicrobial solutions such as bacteriophages. Bacteriophages are viral predators of bacteria, whose actions are almost exclusively specific for their target host species (Clokie et al., 2011; Batinovic et al., 2019). There have been extensive reports and reviews on the application of bacteriophages in clinical scenarios (Genevière et al., 2021, Liu et al., 2021b, Pirnay et al., 2022; Satta et al., 2022). More recently, bacteriophage therapy has been successfully applied in human clinical trials, and on compassionate grounds for critically ill patients with resistant and difficult to treat infections, in Western countries (Vandenheuvel et al., 2015; Schooley et al., 2017; Dedrick et al., 2019; Maddocks et al., 2019). In some instances, these applications have resulted in complete bacterial elimination, a phenomenon not previously reported when using conventional antibiotics (Wunderink, 2019). Bacteriophage therapy has also been shown to be well tolerated and safe when administered intravenously (Petrovic Fabijan et al., 2020). Our previous work has reviewed the role of important oncobacteria, in many cancers, as well as introducing the prospect of using bacteriophages to negate the effects of the pro-oncogenic microbiome (Kabwe et al., 2021a). Although other means of manipulating the microbiome such as antibiotics, probiotics, prebiotics and faecal transplantation (Abdul Rahman et al., 2021) have been considered, all are usually nonspecific and offer varied results. For instance, with antibiotics there are problems such as resistance, and their incapacity to reach therapeutic levels to eliminate intracellular bacteria (Imbuluzqueta et al., 2010) or bacteria in biofilms (Hoiby et al., 2010). Further, they lack specificity for bacteria that promote cancer progression, and contribute to dysplasia (Boursi et al., 2015; Theochari et al., 2018; Dicks et al., 2019). Similarly, probiotics and faecal transplants may contain biological material that do not directly remove oncobacteria and pose the risk of transmitting pathogenic bacteria (Abdul Rahman et al., 2021). Bacteriophages, on the other hand, offer the possibility of precisely eliminating specific bacteria in the microbiome (Belizario and Napolitano, 2015; Clavijo and Florez, 2018; Gagliardi et al., 2018; Paule et al., 2018) and they overcome resistance from bacteria as they have done during co-evolution with their hosts over time (Shabbir et al., 2016).

There has been some research into manipulation of the microbiome in pancreatic cancer. Preclinical data have shown that intratumour bacteria metabolise gemcitabine to its inactive form in pancreatic cancer (Geller et al., 2017; Thomas, 2017; Geller and Straussman, 2018). A recent review of retrospective clinical data of antibiotic use in pancreatic cancer patients did not reveal any significant differences in patients with localised disease but showed significant improvement in survival in metastatic pancreatic patients by at least 2-3 months when patients received gemcitabine-based chemotherapy as first-line therapy (Mohindroo et al., 2021). However, antibiotic use creates clinical problems in pancreatic cancer treatment. A recent analysis of the comparator arm of the MPACT trial (NCT01442974) showed that use of antibiotic therapy was associated with grade 3 or above haematological and gastrointestinal adverse events in patients treated with gemcitabine for metastatic pancreatic cancer (Corty et al., 2020). Scope therefore exists for antimicrobial therapy that targets cancer promoting bacteria and negates chemotherapy resistance and adverse events associated with antibiotic use. Bacteriophage use in cancer therapy is not a new concept, first suggested by Bloch in 1940 (Budynek et al., 2010). Recent studies in this area include the finding that bacteriophage EFA1 was able to eliminate and negate the proliferative effects of *Enterococcus faecalis* on colon cancer cells (Kabwe et al., 2021b). In other work, azodibenzocyclooctyne-modified irinotecan loaded dextran nanoparticles were covalently linked to azide-modified *Fusobacterium* bacteriophages. This construct, a bacteriophage-guided biotic–abiotic hybrid nanosystem, was used to deliver the encapsulated chemotherapy and eliminate intratumour *F. nucleatum* in piglets, without significant changes in haemocyte counts, immunoglobulin and histamine levels, or liver and renal functions (Zheng et al., 2019).

In this study, we reviewed bacteria that have increased relative abundance in patients with pancreatic cancer, including bacteria in the pancreatic tumour microenvironment itself. However, extending the investigation to tissues upstream and adjacent to the pancreas is also important. This is because in many systemic diseases the state of the oral cavity often predicts the health of an individual (Rautemaa et al., 2007), and the majority of oncobacteria are often of oral origin (Kabwe et al., 2021a). Therefore, exploration of the oral microbiome changes in pancreatic cancer is important. Further, due to retrograde bacterial migration from the duodenum to the opening of the pancreatic duct, the microbiome of the duodenum may provide data on predicting pancreatic cancer development (Geller et al., 2017). The microbiome of stools is also commonly assessed. Analysis of the microbiome in these sites provides minimally invasive opportunities for potential diagnosis of pancreatic cancer. At the same time, bacteria whose relative abundance is increased may be specifically targeted by precision antibacterial therapy such as can be offered by bacteriophages. Such possibilities are discussed in this study.

## Methods

We performed a systematic literature review to identify the important bacteria in the microbiome of pancreatic cancer patients. We searched PubMed central (PMC), Medline and Web of Science for studies that compared microbiome composition of pancreatic cancer patients to non-pancreatic cancer patients up to October 2021. We used the Preferred Reporting Items for Systematic Reviews and Meta-Analyses (PRISMA) method, as previously described. (Liberati et al., 2009). The search terms were: (“Fine needle” OR “FNA” OR “FNB”) AND pancreatic microbiome; (“phage” OR “bacteriophage”) AND pancreatic microbiome; Pancreas AND bacteriophages; Pancreas AND microbiome; and Pancreas AND virome. Articles were tracked using Microsoft Excel, removing duplicates and screening firstly by title, then abstract screening and finally screening the full text. All references were managed using the Endnote X9 reference manager.

### Inclusion Exclusion Criteria

The study selection included English-written articles investigating global abundance of different bacteria genera and species across a patient sample in comparison to matched non-pancreatic cancer patients or tissues. The characteristics of studies excluded were: reviews, languages other than English, studies on biomarkers other than microbiome, microbiome in non-cancer/non malignancy, animal studies, abstracts and posters, antibiotic use, bacterial species-specific PCR amplification studies, studies describing phyla only, studies without non-malignant controls.

## Results

### Studies Identified

**Figure 2:**
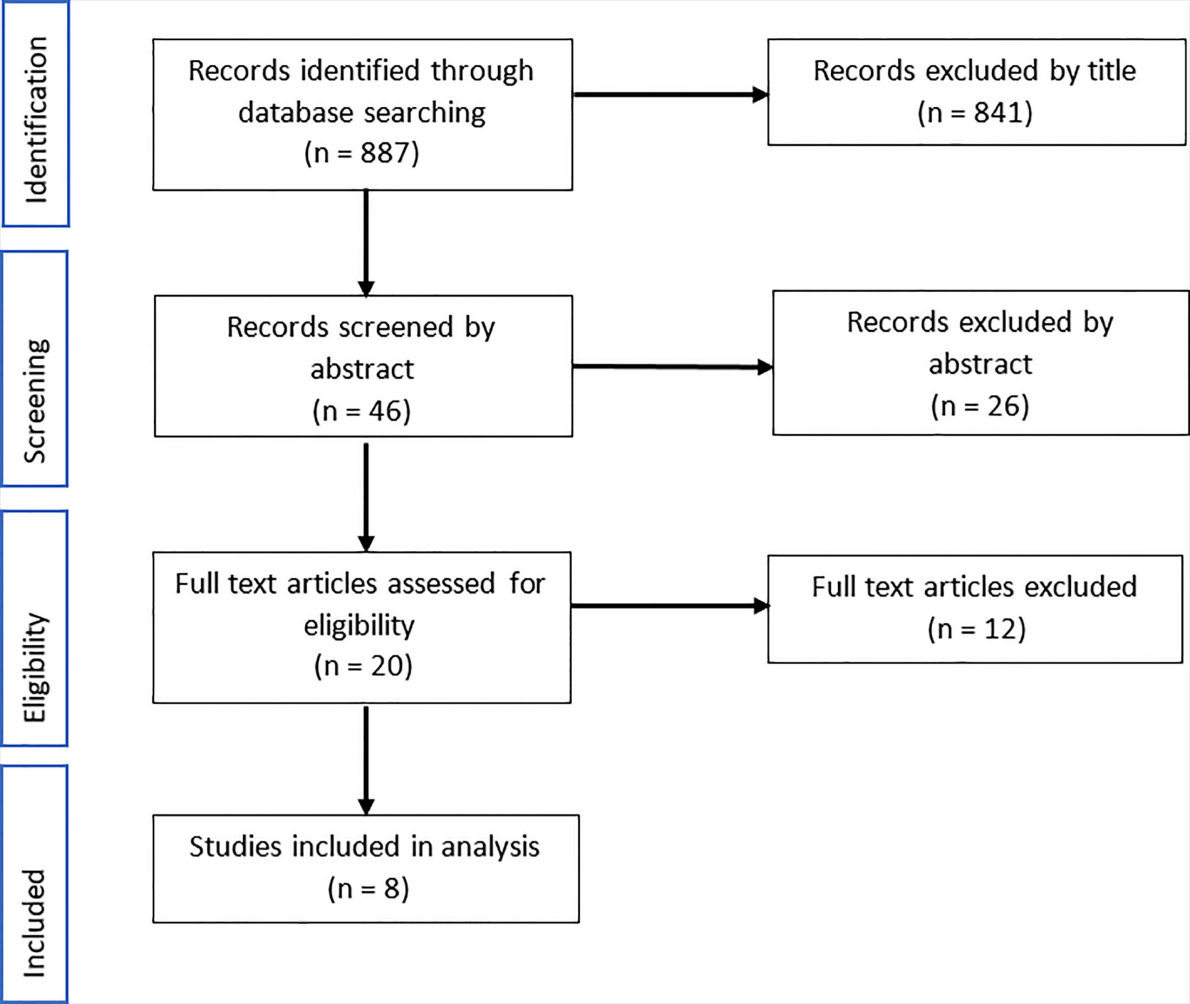
Flow diagram of documents selection for review on identifying bacteria whose relative abundance was significantly different in pancreatic cancer patients compared with healthy normal controls. Records were sourced from Medline, PMC and Web of Science up to October 2021.

A total of 887 articles were identified from the search strategy. The initial title screening eliminated 841 articles to leave 46 for abstract assessment. From the abstracts, 26 were eliminated based on the exclusion criteria and a further 12 articles were excluded on examination of the full text, leaving eight articles to be incorporated into the analysis (**Figure 2**).

Some of the studies that were reviewed undertook prospective recruitment of participants while others utilised tissue samples that were available in their institutional biobanks. Only one study reported both the number of participants and number of samples examined (Del Castillo et al., 2019): the authors recruited 76 participants, whose ethnicities were described as Caucasian (72), black (2) and other (2). Among these participants, there were 50 patients with pancreatic cancer. The authors examined 189 tissue samples (pancreatic duct, duodenum, pancreas), 57 swabs (bile duct, jejunum, stomach), and 12 stool samples (Del Castillo et al., 2019) (**Table 1**).

**Table 1:** Results from studies investigating bacterial relative abundance in pancreatic tumour patients and pancreatic tumour samples.

Bacterial genera increased in relative abundance	Sites where bacterial genera were increased in relative abundance	Study type and size	Population	Reference
*Porphyromonas*	Pancreas, Pancreatic duct	189 tissue samples (pancreatic duct, duodenum, pancreas), 57 swabs (bile duct, jejunum, stomach), and 12 stool samples	**Case**: Caucasian 72 (93.5%), Black 2 (2.6%), Others 2 (2.6%) **Control**: Caucasian 30 (88%), Black 2 (6%), Others 2 (6%) **Cases**: Male 38 (49%); Female 39 (51%) **Controls**: Male 21 (62%); Female 13 (38%)	(Del Castillo et al., 2019)
*Fusobacterium, Prevotella, Capnocytophaga, Parvimonas*, and *Haemophilus*	Pancreas			
*Selenomonas*	Pancreas, Duodenum			
*Gemella*	Duodenum			
*Fusobacterium, Enterobacter*, *Citrobacter*, and *Klebsiella*	Pancreas	Studied 1526 tumours and their adjacent normal tissues across seven cancer types, including breast, lung, ovary, pancreas, melanoma, bone, and brain tumours. (67 pancreatic tumour tissues)	Biobank	(Nejman et al., 2020)
*Fusobacterium*	Pancreas	Formalin Fixed, paraffin embedded samples from 17 patients were studied; 9 of whom had pancreatic cancer All 17 had EUS-FNB samples and 6 pancreatic cancer cases had paired resection samples.	Participant numbers listed only	(Masi et al., 2021)
*Acinetobacter, Aquabacterium, Oceanobacillus, Rahnella*, *Massilia, Delftia app., Deinococcus*, and *Sphingobium*	Duodenum	In this study, duodenal mucosal microbiota was analysed in 14 patients with pancreatic head cancer and 14 healthy controls	Participant numbers listed only	(Mei et al., 2018)
*Bifidobacterium, Fusobacterium* *Rothia, Enterococcus, “Escherichia-Shigella”*, and *Clostridium*	Duodenum	Case-control study comparing bacterial and fungal (16S and 18S rRNA) profiles of secretin-stimulated duodenal fluid collections from 308 patients undergoing duodenal endoscopy including 134 normal pancreas control subjects, 98 patients with pancreatic cyst (s) and 74 patients with pancreatic cancer.	**Cases**: White 57 (90.4%), African American 4 (6.4%), missing 2 (3.2%) **Controls**: White 57 (90.4%), African American 5 (8%), Missing 1 (1.6%) **Cases**: Male 40 (63.5%), Female 23 (36.5%) **Controls**: Male 30 (47.6%), Female 33 (52.4%)	(Kohi et al., 2020)
*Porphyromonas, Aggregatibacter*, and *Alloprevotella*	Oral	361 incident adenocarcinoma of pancreas and 371 matched controls were selected from two prospective cohort studies, the American Cancer Society Cancer Prevention Study II and the National Cancer Institute Prostate, Lung, Colorectal, and Ovarian Cancer Screening Trial.	**Cases**: White 338 (93.6%), Non-White 23 (6.4%) **Controls**: White 345 (93.0%), Non-White 26 (7%) **Cases**: Male 206 (57.1%), Female 155 (42.9%) **Controls**: Male 212 (57.1%), Female 159 (42.9%)	(Fan et al., 2018)
*Actinomyces, Aggregatibacter*, *Escherichia Lactobacillus, Leptotrichia, Rothia, and Streptococcus*	Oral	Prospective study of saliva samples collected from patients with pancreatic cancer (n = 41) and healthy individuals (n = 69).	**Cases**: Male 24 (59%), Female 17 (41%) **Controls**: Male 52 (72%), Female 19 (28%)	(Wei et al., 2020)
*Cronobacter*, *Enterobacter*, * (* Ren et al., 2017*) Hallella*, * (* Ren et al., 2017*) Klebsiella, Prevotella*, *Selenomonas*, and *Veillonella*	Stool	Prospective study that collected 85 Pancreatic cancer and 57 matched healthy controls to analyze microbial characteristics by MiSeq sequencing	Cases: Male 47 (55.3%); Female 38 (44.7%) Control: Male 36 (63.2); Female 21 (36.8%)	(Ren et al., 2017)

Another American report described a case-control study comparing bacterial profiles from 63 pancreatic cancer patients and 63 healthy controls, with 57 in each group denoted as Caucasian, as well as five African Americans and one unknown in the healthy participants; four African Americans and two unknown in the patient cohort (Kohi et al., 2020). Other studies that listed the number of participants were from the United Kingdom (UK) and China, however, ethnicity of the participants wasn’t noted. The UK study recruited 17 participants of which nine had pancreatic cancer (Masi et al., 2021). There were three Chinese studies; Mei et al. recruited nine pancreatic cancer patients and eight controls (Mei et al., 2018), Ren et al, collected 85 pancreatic cancer patients and 57 matched healthy controls (Ren et al., 2017) while the other study recruited 80 patients and 69 healthy controls from the community (Wei et al., 2020) (**Table 1**).

The remaining two studies utilised biobanks and analysed large numbers of samples. These included 361 samples of incident adenocarcinoma of the pancreas and 371 matched controls, from two prospective cohort studies: the American Cancer Society Cancer Prevention Study II and the National Cancer Institute Prostate, Lung, Colorectal, and Ovarian Cancer Screening Trial (Fan et al., 2018). The other study to utilise biobanks analysed 1526 samples across seven cancer types (tumours and their adjacent normal tissues), of which 67 were from pancreatic cancer patients (Nejman et al., 2020) (**Table 1**).

### Bacteria With Significantly Increased Relative Abundance

**Figure 3:**
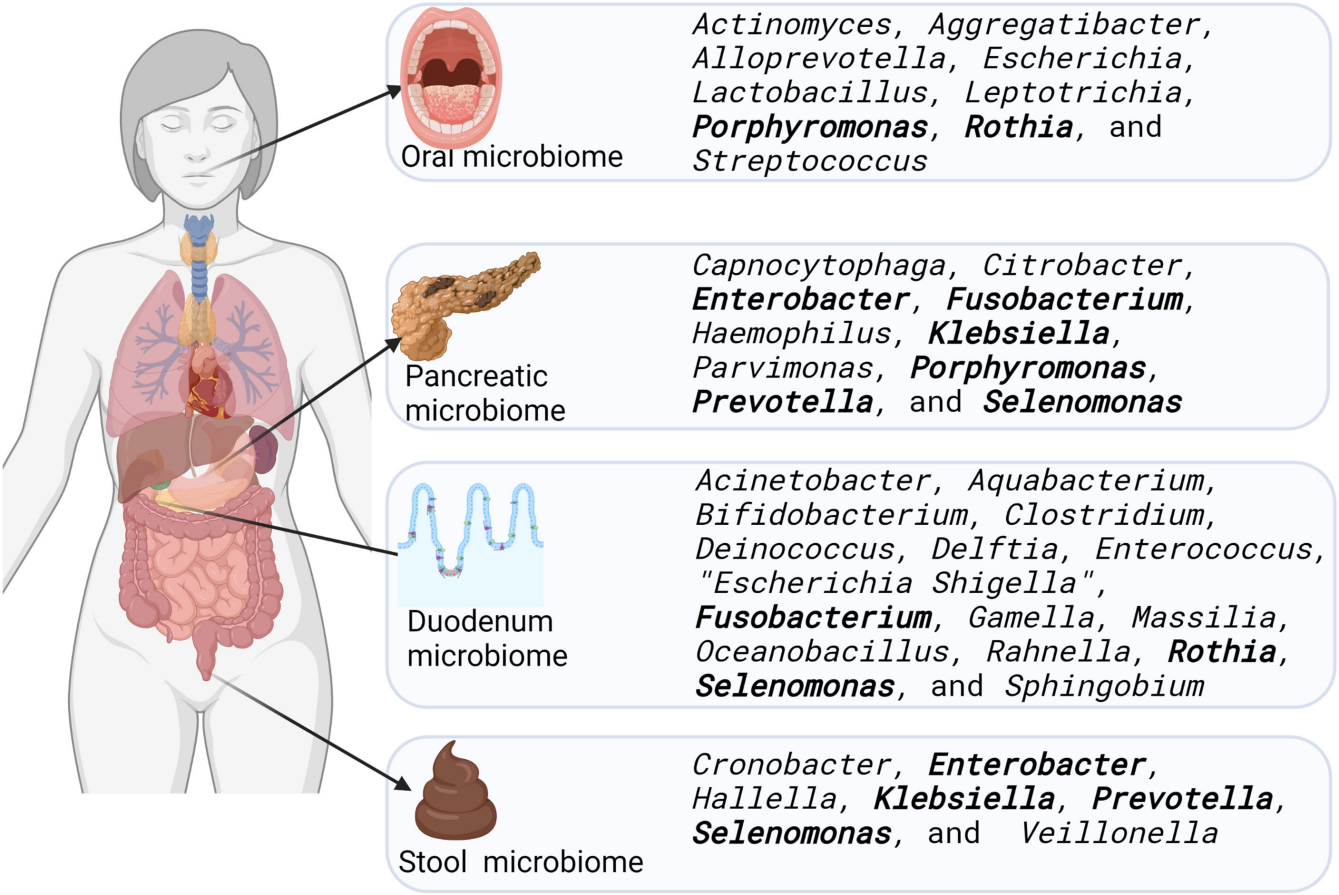
Bacterial genera increased in relative abundance in the microbiome of the oral cavity, pancreas, duodenum, and stools of pancreatic cancer patients, compared to samples from people without pancreatic cancer. Bacteria whose relative abundance were increased in more than one site are highlighted in bold font. Created in Biorender.com

Eight studies were identified as having compared microbiome sequence data from pancreatic cancer patients with that from non-pancreatic cancer patients. These studies compared the microbiomes of the oral cavity, pancreas, duodenum, and stools (**Figure 3**). They identified specific bacteria in the tissues examined to be a signature of pancreatic cancer, and our review collated this data, and indicated that a range of bacteria had their relative abundances increased in this disease. Our findings identified seven bacterial genera whose relative abundance was increased in more than one anatomic site (highlighted in bold in **Figure 3**). *Selenomonas* were increased in three of four sites reviewed including the pancreas (Del Castillo et al., 2019), the duodenum (Del Castillo et al., 2019) and stools (Ren et al., 2017). *Porphyromonas* were increased in the pancreas (Del Castillo et al., 2019) and oral cavity (Fan et al., 2018). *Fusobacterium* were increased in the pancreas (Del Castillo et al., 2019; Nejman et al., 2020; Masi et al., 2021) and the duodenum (Kohi et al., 2020). *Enterobacter*, *Klebsiella* and *Prevotella* were all increased in the pancreas (Nejman et al., 2020) and stools (Ren et al., 2017), while *Rothia* were increased in the duodenum (Kohi et al., 2020) and the oral (Wei et al., 2020) microbiomes.

## Discussion

### Established Role of Keystone Bacteria Identified in Pancreatic Cancer Microbiomes

It has been proposed that oral bacteria may play a role in the causation of cancer locally and in distant organs, as these oncobacteria may traverse impaired blood vessels and circulate to colonise distant tissues where they promote cancer growth (Kostic et al., 2013; Abed et al., 2020; Kabwe et al., 2021a). For instance, it has been shown using murine models that *Fusobacterium* normally resident in gingival margins and the crypts of the tongue may spread through the circulatory system (Abed et al., 2020) as well as through the gastrointestinal tract (Kostic et al., 2013; Pushalkar et al., 2018) to the pancreas. As such, it may be expected that a particular bacterial species may be located in more than one of the oral, pancreatic, duodenal, and faecal microbiomes in cancer patients. In this systematic review of the literature, we identified seven bacteria genera whose relative abundance was increased in more than one of these anatomic sites (**Figure 3**). In the pancreas and oral cavity, *Porphyromonas* was increased; in the pancreas and the duodenum, *Fusobacterium* and *Selenomonas* were increased, while in the pancreas and stool, *Enterobacter*, *Klebsiella*, *Prevotella* and *Selenomonas* were all increased. *Selenomonas* was the only bacterial genus reported to have increased abundance in at least three of four sites explored. *Rothia* was the only bacterial genus that were increased in more than one site excluding the pancreas. These were increased in the duodenum and the oral microbiomes (**Figure 3**).

**Figure 4:**
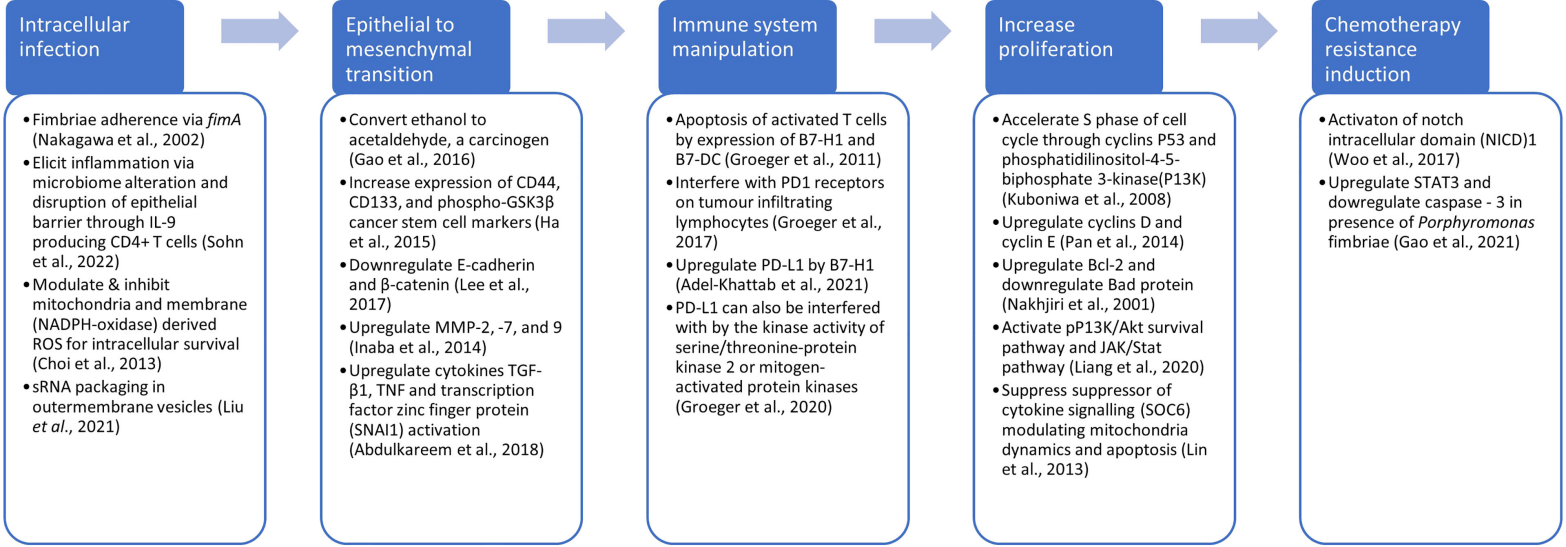
Possible mechanisms for promotion of carcinogenesis by *Porphyramonas*, including infection, epithelial to mesenchymal transition, evasion of immune system, increased proliferation and chemotherapy resistance.

While microbiome sequencing studies are important in identifying potentially oncogenic bacteria, experimental evidence remains key to demonstrating their effects on cancer progression. It is also important to highlight that there are complex interactions within tissue microbiomes which may influence the initiation of cancers. Within this polymicrobial ecosystem there may be species that while not directly oncogenic, may promote the colonisation of a site by an oncobacterium, or others that protect bacteria from the host response. Our previous review on tissue microbiomes across several cancer types has revealed a range of different bacteria whose relative abundance was shown to be increased or decreased in the tumour microbiome, as well as those shown to attenuate, as well as promote oncogenicity (Kabwe et al., 2021). *Fusobacterium* is the most studied oncobacteria in diverse cancer types where mechanisms of oncogenicity have been elucidated (Slade, 2021). In pancreatic cancer, *Porphyromonas gingivalis* is the most studied, and data have shown in murine models that they are able to promote progression of pancreatic cancer (Chen et al., 2020; Gnanasekaran et al., 2020; Hiraki et al., 2020). Other experimental data have shown that the *P. gingivalis* may invade and promote proliferation of other cancer types including oral squamous cell carcinoma (Inaba et al., 2014; Geng et al., 2017; Chang et al., 2019; Hoppe et al., 2019; Yao et al., 2021), endothelial cancer (Crooks et al., 2021), colorectal carcinoma (Mu et al., 2020; Wang et al., 2021), oesophageal squamous cell carcinoma (Meng et al., 2019; Liang et al., 2020; Chen et al., 2021), and head and neck cancer (Utispan et al., 2018). Further, *P. gingivalis* has been shown to promote chemotherapy resistance to paclitaxel in oral squamous cell carcinoma (Song et al., 2019). The mechanisms by which *P. gingivalis* promotes gastrointestinal cancers including pancreatic cancer have been reviewed (Olsen and Yilmaz, 2019). As shown in **Figure 4**, its role in cancer commences with infection of epithelial cells, with the FimA fimbria as the main adhesion and invasive virulence factor (Nakagawa et al., 2002). This induces inflammation and disruption of the epithelial barrier through IL-9 induced CD4+ T cells (Sohn et al., 2022) whilst enhancing its intracellular survival through modulating reactive oxygen species production (Choi et al., 2013) and small RNA packaging in outer membrane vesicles (OMVs) (Liu et al., 2021a). Further*, P. gingivalis* has been shown to induce carcinogenesis by epithelial to mesenchymal transition (Inaba et al., 2014; Ha et al., 2015; Gao et al., 2016; Lee et al., 2017; Abdulkareem et al., 2018) and to promote cancer proliferation (Nakhjiri et al., 2001; Kuboniwa et al., 2008; Lin et al., 2013; Pan et al., 2014; Liang et al., 2020). *P. gingivalis* has also been shown to modulate the immune system, enabling survival of the tumours (Groeger et al., 2011; Groeger et al., 2017; Groeger et al., 2020; Adel-Khattab et al., 2021), as well as promoting chemotherapy resistance (Woo et al., 2017; Gao et al., 2021).

Apart from *P. gingivalis* however, there is no knowledge of how the other bacteria may influence the progression of pancreatic cancer. For some, there is evidence of the role they play in other cancer types (**Table 2**). *F. nucleatum* can travel *via* the blood stream (Abed et al., 2020) and lodge into distant tumours of the breast (Hieken et al., 2016; Smith et al., 2019), lung (Liu et al., 2018) and genitourinary tract (Audirac-Chalifour et al., 2016), where they colonise nascent tumours (Cavrini et al., 2005) or promote metastasis (Parhi et al., 2020). Further, tumours with an increased abundance of *F. nucleatum* have been associated with poor prognosis and chemotherapy resistance (Yamamura et al., 2016; Bullman et al., 2017; Kunzmann et al., 2019). Bacterial extracts from *Enterobacter* have been shown to inhibit myeloid leukemia HL-60 cells (Husain et al., 2016) and cervical cancer HeLa and SiHa cells (Zhao et al., 2015), as well as promoting NCM460 and CRL1700 colon cancer cells (Yurdakul et al., 2015). *Prevotella* have also displayed contrasting actions with *P. heparinolytica* shown to indirectly promote multiple myeloma while *P. melaninogenica* counters such effects (Calcinotto et al., 2018). Some *Klebsiella* species have been linked to cancer cachexia (Pötgens et al., 2018) and their production of colibactin induces tumourigenic effects in epithelial cells (Strakova et al., 2021). The other bacteria increased in the pancreatic tumour microenvironment included *Capnocytophaga* (Del Castillo et al., 2019), *Citrobacter* (Nejman et al., 2020), *Haemophilus* (Del Castillo et al., 2019), and *Parvimonas* (Del Castillo et al., 2019). Of these, *Citrobacter rodentium* has been shown to increase the proliferation of colon cancer and to activate cancer promoting biochemical pathways (Umar, 2012), and *Haemophilus influenzae* has been shown to increase the proliferation of k-ras positive lung adenocarcinoma (Jungnickel et al., 2017). There were no studies of *Parvimonas* or *Capnocytophaga* roles in cancer, although their prevalence has been reported to be increased in several different tumour microenvironments (Jolivet-Gougeon and Bonnaure-Mallet, 2021; Kabwe et al., 2021a). No research detailing the role of *Selenomonas* or *Rothia* in cancer progression has been reported either.

**Table 2:** Potential significance of the bacteria whose relative abundance are increased in pancreatic cancer.

Bacteria genera	Possible significance in cancer
*Porphyromonas*	*Porphyromonas gingivalis* has been shown to promote progression of pancreatic cancer (Chen et al., 2020; Gnanasekaran et al., 2020; Hiraki et al., 2020), oral squamous cell carcinoma (Inaba et al., 2014; Geng et al., 2017; Chang et al., 2019; Hoppe et al., 2019; Yao et al., 2021), endothelial cancer (Crooks et al., 2021), colorectal carcinoma (Mu et al., 2020; Wang et al., 2021), oesophageal squamous cell carcinoma (Meng et al., 2019; Liang et al., 2020; Chen et al., 2021), and head and neck cancer (Utispan et al., 2018). promote chemotherapy resistance to paclitaxel in oral squamous cell carcinoma (Song et al., 2019).
*Fusobacterium*	Can travel *via* the blood stream (Abed et al., 2020) and lodge into distant tumours of the breast (Hieken et al., 2016; Smith et al., 2019), lung (Liu et al., 2018) and genitourinary tract, (Audirac-Chalifour et al., 2016) where they colonise nascent tumours (Cavrini et al., 2005) or promote metastasis (Parhi et al., 2020) Tumours with an increased abundance of *F. nucleatum* have been associated with poor prognosis and chemotherapy resistance (Yamamura et al., 2016; Bullman et al., 2017; Kunzmann et al., 2019).
*Enterobacter*	Inhibit myeloid leukemia HL-60 cells (Husain et al., 2016) and cervical cancer HeLa and SiHa cells (Zhao et al., 2015), as well as promoting NCM460 and CRL1700 colon cancer cells (Yurdakul et al., 2015).
*Prevotella*	*Prevotella heparinolytica* promote multiple myeloma while *Prevotella melaninogenica* counters such effects (Calcinotto et al., 2018).
*Klebsiella*	Klebsiella sp. Infection has been linked to cancer cachexia (Pötgens et al., 2018) and their production of colibactin, induces tumourigenic effects in epithelial cells (Strakova et al., 2021).
*Citrobacter*	*Citrobacter rodentium* has been shown to increase the proliferation of colon cancer and to activate cancer promoting biochemical pathways (Umar, 2012)
*Haemophilus*	*Haemophilus influenzae* has been shown to increase the proliferation of k-ras positive lung adenocarcinoma (Jungnickel et al., 2017).
*Enterococcus*	*Enterococcus faecalis* has been shown to promote proliferation of colon cancer cells (Kabwe et al., 2021b).
“*Escherichia-Shigella*”	*Polyketide synthetase* (pks) positive *E. coli* strains that express the afimbrial adhesin operon afaC and/or the polyketide synthetase pathogenic island have been shown to promote tumuorigenesis of colon cancer (Lennard et al., 2016, Iyadorai et al., 2020).
*Deinococcus*	Extracts from *Deinococcus* sp. UDEC-P1 have been shown to inhibit the proliferation of Saos-2 osteosarcoma cells (Tapia et al., 2021) and breast cancer (Maqbool et al., 2020).
*Bifidobacterium*	Shown to have anti-proliferative effects against colon (Lee et al., 2008; Asadollahi et al., 2020; Faghfoori et al., 2021) and non-small cell lung cancers (An et al., 2020)
*Clostridium*	*Clostridium perfringens* have been shown to inhibit proliferation of colon cancer cells (Arimochi et al., 2006)
*Parvimonas*	No clear role established
*Capnocytophaga*	No clear role established
*Selenomonas*	No clear role established
*Rothia*	No clear role established
*Acinetobacter*	No clear role established
*Aggregatibacter*	No clear role established
*Alloprevotella*	No clear role established
*Aquabacterium*	No clear role established
*Delftia*	No clear role established
*Gemella*	No clear role established
*Massilia*	No clear role established
*Oceanobacillus*	No clear role established
*Rahnella*	No clear role established
*Sphingobium*	No clear role established

### Implications for Diagnosis

Although the oral, duodenal and faecal microbiomes have been previously suggested as potential biomarkers in pancreatic cancer (Sun et al., 2020), our study has shown a diversity in the microbiome across these sites. For this reason, it may be difficult to select specific bacteria to use as biomarkers in these sites for application in pancreatic cancer diagnosis. Further, while some studies have identified bacteria to be increased solely in cancer patients, others have found increases also in non-cancer controls, or that their prevalence is not consistently increased in all tissues assayed. For instance, *Porphyromonas* were reported to have been increased in the duodenum (Mei et al., 2018) and oral microbiome (Wei et al., 2020) of healthy controls, and *Selenomonas*, *Prevotella* (Wei et al., 2020) and *Fusobacterium* (Fan et al., 2018) were all decreased in the oral microbiome of pancreatic cancer patients. It may be that the faecal, oral and duodenal microbiomes may not represent ideal biomarkers for pancreatic cancer. Further, they may not be specific enough as similar patterns across oral, gut, and intratumour microbiomes may be observed in many different tumour types (Kabwe et al., 2021a; Stasiewicz et al., 2021). As such, screening of the oral, duodenal or faecal microbiomes may not provide an accurate and specific diagnostic tool for pancreatic cancer. In contrast, the pancreatic tumour tissue microbiome may have diagnostic potential. However, more work is needed to establish this, as there is limited data to date. Other indirect serological methods may be considered, for instance, Michaud et al. (Michaud et al., 2013) found that individuals with high levels of circulating antibodies against *P. gingivalis*, had a significant two-fold higher risk of pancreatic cancer than individuals with lower levels of these antibodies (OR 2.14; 95% CI 1.05 to 4.36).

### Potential for Targeting Keystone Bacteria Using Bacteriophage Therapy

**Figure 5:**
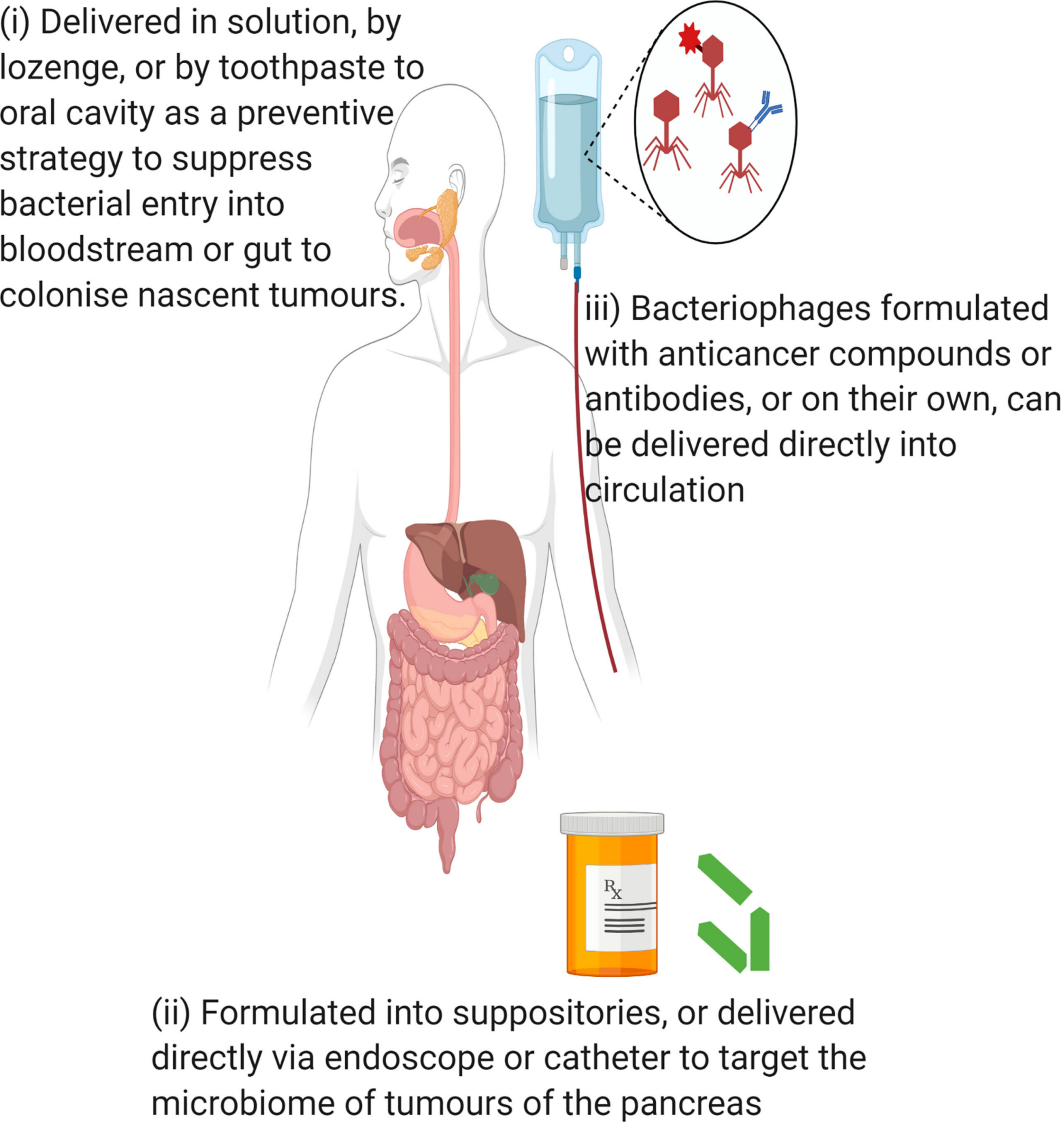
Possible methods of bacteriophage delivery for manipulation of pancreatic cancer microbiome. Created in Biorender.com.

Bacteriophages have been shown to have the capacity to disrupt biofilms (Lusiak-Szelachowska et al., 2020) and eliminate intracellular bacteria (Goswami et al., 2021). One key feature by which bacteria such as *P. gingivalis* promote their spread and colonisation is through outer membrane vesicles (OMVs) (Farrugia et al., 2020). OMVs increase the permeability of blood vessels allowing for systemic spread of the bacteria to colonise distant organs (Farrugia et al., 2020; Zhang et al., 2021) and such a mechanism may allow *P. gingivalis* to contribute to pancreatic cancer. It has also been suggested that OMVs may provide defence mechanisms, in that they act as decoys for bacteria to lure bacteriophages and the immune system in order to limit their effectiveness against the bacterial community (Manning and Kuehn, 2011; Reyes-Robles et al., 2018; Furuyama and Sircili, 2021). As such, prudent application of bacteriophages, such as in suppressing the bacterium in the oral cavity, may be warranted for potential therapy or prophylaxis against *P. gingivalis* in cancers. Bacteriophages also have the capacity to modulate the immune system (Van Belleghem et al., 2018; Popescu et al., 2021) which is an aspect being explored as a novel treatment for pancreatic cancer (Krishnamoorthy et al., 2020). Bacteriophages have been used to treat pancreatitis caused by multidrug resistant *Acinetobacter baumanni* (Schooley et al., 2017), indicating their capacity to treat invasive bacterial infection in this organ. To date, there have been no studies that have evaluated the use of bacteriophages for treatment of pancreatic cancer or modulation of its microbiome. Possible methods for delivery of bacteriophage have been summarised in **Figure 5**.

As shown in **Figure 3**, a range of bacteria genera have been identified with increased abundance in the pancreatic tumour microbiome. Of these, the potential exists for precision control of *Klebsiella*, as numerous bacteriophages against this genus have been isolated and fully characterised (Herridge et al., 2020). *Fusobacterium* have had only one lytic bacteriophage isolated and fully characterised (Kabwe et al., 2019). Other lytic bacteriophages against *F. nucleatum* (Machuca et al., 2010) and *F. necrophorum* (Tamada et al., 1985), as well as prophages against *Fusobacterium varium* (Andrews et al., 1997) and *Fusobacterium symbiosum* (Foglesong and Markovetz, 1974), have been described but not fully characterised. Lytic bacteriophages that target *Citrobacter rodentium* and *Citrobacter freundii* have also been isolated and characterised (Chaudhry et al., 2014; Edwards et al., 2015; Hwang et al., 2015; LeSage et al., 2015; Nguyen et al., 2015; Shaw et al., 2015; Tikhe et al., 2015; Hamdi et al., 2016; Mizuno et al., 2020). For *H. influenzae* and *Haemophilus parasuis* only prophages have been isolated and characterised (Jablońska et al., 1976; Williams et al., 2002; Zehr et al., 2012; Adamczyk-Popławska et al., 2020). To date there have been no bacteriophages found against *Porphyromonas*, *Capnocytophaga*, *Enterobacter*, *Parvimonas*, *Prevotella*, or *Selenomonas*. Bacteriophages are the most abundant organisms in the human body (Cani, 2018) and possibly in nature (Clokie et al., 2011), so efforts to isolate bacteriophages that target oncogenic bacteria are warranted. Metagenomic analysis of the phageome in the oral cavity has revealed an extensive resource that may yield suitable bacteriophages (Szafrański et al., 2019; Szafrański et al., 2021). No studies as yet have focussed on the pancreatic phageome.

## Conclusion

We have identified some bacteria that need to be further investigated regarding their contribution to carcinogenesis in pancreatic cancer. Of these, we could only find experimental evidence that supports promotion of pancreatic cancer growth by *Porphyromonas*. There is need for more primary data to examine the role of other possible oncobacteria in the development and progression of pancreatic cancer. There is evidence that bacteriophages can access and treat multi-antibiotic resistant pancreatic infections. Studies into their role in pancreatic cancer or their capacity to manipulate the pancreatic tumour microbiome should accompany those elucidating the role of oncobacteria in these tumours. However, this will only be possible if more bacteriophages against these keystone bacteria are isolated.

## Author Contributions

MK and JT, conceptualization. MK, JT, and SD, data search. MK, JT, and SD, draft manuscript. MK, JT, and SD, finalization of manuscript. All authors contributed to the article and approved the submitted version.

## Conflict of Interest

The authors declare that the research was conducted in the absence of any commercial or financial relationships that could be construed as a potential conflict of interest.

## Publisher’s Note

All claims expressed in this article are solely those of the authors and do not necessarily represent those of their affiliated organizations, or those of the publisher, the editors and the reviewers. Any product that may be evaluated in this article, or claim that may be made by its manufacturer, is not guaranteed or endorsed by the publisher.
